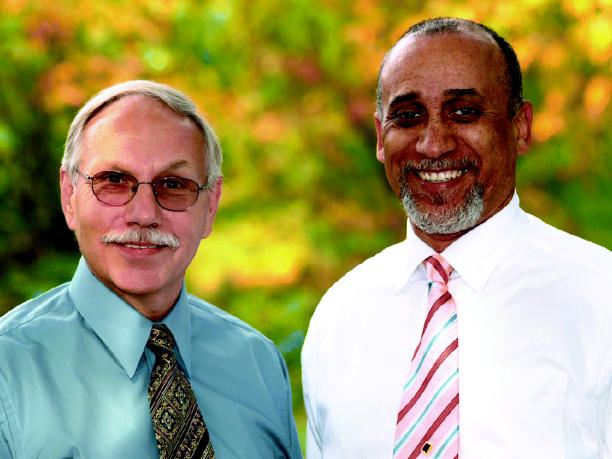# Environmental Influences on Epigenetic Regulation

**Published:** 2005-12

Epigenetics refers to the study of gene silencing and non-Mendelian inheritance of traits. By controlling the expression of key genes and associated pathways, epigenetics provides an elegant mechanism for time and tissue-specific expression of key biologic molecules across the life span.

The available data support the notion that epigenetic processes can be influenced by both exogenous and endogenous factors. Specific agents in the areas of nutrition, environmental chemicals, stressors, and pharmaceutical agents have been shown to affect the epigenetic state. Few data are available to address the potential relationship between such changes and adverse functional outcomes. The role of epigenetics in the regulation of normal development as well as the impact of exogenous influences (e.g., environmental chemicals, stressors, nutrition, drugs) on epigenetic pathways is an emerging area of interest.

The National Institute of Environmental Health Sciences (NIEHS) is developing a comprehensive program that addresses how gene expression is altered under different forms of environmental stress and how this alters the risk of developing disease.

Two recent events signal the role NIEHS is undertaking to increase support of research to enhance understanding of how environmental perturbations occurring throughout the life span can affect phenotypes at different stages of life and/or transgenerationally by altering gene expression though modification of DNA methylation and chromatin structure. First, on November 2–4, in collaboration with Duke University Medical Center, NIEHS co-sponsored an international symposium titled *Environmental Epigenomics, Imprinting and Disease Susceptibility* (**http://www.geneimprint.com/meetings/2005durham/**). Second, as part of a series of solicitations to be released over the next 5 years, NIEHS announced RFA ES-05-007, *Environmental Influences on Epigenetic Regulation*, to support research on environmental stressor effects on gene silencing in somatic cells and altered methylation profiles that are related to disease outcomes at various stages of the life span.

These solicitations are anticipated to stimulate research directed toward the understanding of the role of epigenetic modulation of gene expression in a variety of diseases and dysfunctions and the modulation of epigenetic regulation by environmental chemicals either alone or in combination with altered nutrition. Other areas of emphasis could include understanding the role of epigenetics in the transgenerational effects of environmental agents, the role of environmental stressors in the suppression of gene expression, the identification of epigenetic targets in the genome sensitive to environmental modification, and the mechanism of interaction of environmental chemicals with potential modifiers of epigenetic processes.

## Contact

**Frederick L. Tyson, PhD** | tyson2@niehs.nih.gov

**Jerrold Heindel, PhD** | heindelj@niehs.nih.gov

## Figures and Tables

**Figure:**